# Identification of new candidate genes for the hereditary predisposition to uveal melanoma: IGCMU trial

**DOI:** 10.3389/fonc.2025.1538924

**Published:** 2025-01-24

**Authors:** Mélanie Godiveau, Angeline Ginzac, Yannick Bidet, Flora Ponelle-Chachuat, Maud Privat, Xavier Durando, Mathias Cavaillé, Mathis Lepage

**Affiliations:** ^1^ Université Clermont Auvergne, Institut National de la santé et de la recherche en innovation (INSERM), Unité mixte de recherche (UMR) 1240 « Imagerie Moléculaire et Stratégies Théranostiques », Center Jean Perrin, Clermont-Ferrand, France; ^2^ Division de Recherche Clinique, Délégation Recherche Clinique and Innovation, Center Jean Perrin, Clermont-Ferrand, France; ^3^ Centre d’Investigation Clinique (CIC), UMR501, Clermont-Ferrand, France; ^4^ Département d’Oncogénétique, Laboratoire d’Oncologie Moléculaire, Center Jean Perrin, Clermont-Ferrand, France; ^5^ Département d’Oncologie Médicale, Center Jean Perrin, Clermont-Ferrand, France

**Keywords:** uveal melanoma, germline mutations, exome sequencing, hereditary predisposition, clinical trial

## Abstract

**Introduction:**

Uveal melanoma (UM) is a rare ocular cancer. While germline mutations in genes such as BAP1 and MBD4 account for approximately 20% of familial UM cases, the hereditary factors underlying the remaining cases remain unknown. Epidemiological studies have suggested an increased risk of prostate cancer, thyroid cancer, and leukemia among patients with UM, indicating potential unidentified genetic predispositions. This study aims to identify new candidate genes associated with a hereditary predisposition to UM.

**Methods:**

This single-center study, conducted at Centre Jean Perrin, will involve the exome sequencing of 50 patients with UM who do not harbor known pathogenic variants in the BAP1 or MBD4 genes. The primary objective is to identify novel candidate genes associated with hereditary cancer predisposition among UM patients. A several-step-bioinformatic analysis will be conducted to identify the genes of interest. A secondary objective is to explore genes known to be involved in predisposition to other cancers, already described in the occurrence of uveal melanoma, but where an association has not been fully established yet. The study has begun in October 2024, with patient recruitment lasting 12 months. No follow-up period is planned, but the duration of the genetic analyses is estimated at six months, with the final study report expected by October 2026.

**Discussion:**

The identification of novel hereditary predisposition genes for UM could significantly enhance genetic counselling and surveillance strategies for families affected. This study could also contribute to a better understanding of the genetic landscape of UM, potentially leading to more personalized and effective options for its detection.

**Trial registration:**

ClinicalTrials.gov, identifier NCT06550674, registered in August 2024. Protocol: version 1.0, January 18^th^, 2024.

## 1 Introduction

Uveal melanoma (UM) is a rare, aggressive ocular cancer occurring in adults, with an incidence of 1 to 9 cases per million people per year (1). It arises from melanocytes in the uveal tract, which includes the choroid, the ciliary body, and the iris. The most common symptoms are blurred or distorted vision, visual field loss or photopsia (2, 3). The 5-year survival rates are quite encouraging for localized and regional stages, standing at 85% and 67% respectively. However, the most significant challenge in managing this disease occurs in the metastatic stage, where the survival rate drops to 16% (4). With more than 30% of these patients developing metastases, almost invariably in the liver, UM is therefore known for its poor prognosis as a result of the absence of efficacious treatments, with a median survival of only 10 months (5–7).

Most patients are diagnosed with UM between the ages of 50 and 70 (2). The main risk factors — such as skin phototype, geographic gradient from south to north (linked to the phototype), congenital ocular melanocytes, family history, and hereditary cancer predisposition — are non-modifiable. Focusing on the decreasing north-south gradient, Virgili et al., supported the protective role of skin pigmentation between the population of northern and southern Algeria (8). However, even if UM incidence increase with latitude, there is no evidence for a link between ultraviolet radiation and UM occurrence (8, 9). These factors suggest the important role that genetic predisposition plays in the development of UM (2). Approximately 1 to 2% of UM and 22% of familial cases have been linked to hereditary cancer predisposition and result from pathogenic genetic variations, primarily in the BAP1 gene, and more recently in MBD4. These genes are associated with syndromic hereditary cancer predisposition. Pathogenic variants in BAP1 confer a high risk of various cancers, including UM (16-36% risk), with a median onset age of 53 years compared to 62 years for sporadic forms. This risk extends to other tumors such as cutaneous melanoma, pleural melanoma (20-25%), and renal cancer (10%). MBD4 for its part has been associated with an increased risk of colorectal cancer and acute myeloid leukemia (10). The transmission risk for the BAP1 and MBD4 genes is estimated at 50% per conception, owing to their autosomal dominant inheritance pattern, affecting both males and females. Identifying a pathogenic variant in the BAP1 or MBD4 genes enables tailored medical management for affected individuals and their relatives, including international screening recommendations for BAP1, which involve clinical skin examinations, eye fundus examinations, and imagery for pulmonary and renal assessments (11). Additionally, constitutional genetic anomalies in genes such as BRCA1, BRCA2, CHEK2, PALB2, SMARCE1, POT1, TP53AIP1, MSH6, MLH1, MLH2 and PMS2 have been described in UM patients, although their involvement remains unclear (12). Epidemiological studies have also shown an increased risk of prostate cancer, thyroid cancer, and leukemia among UM patients, which is not currently associated with BAP1 or MBD4 (13, 14).

The identification of candidate genes for hereditary predisposition to uveal melanoma is of great interest, as it holds the potential to revolutionize patient care. Indeed, validation of these potential genes could enable their integration into a diagnostic approach. Patients could then be offered screening and preventive measures tailored to their tumor spectrum, as well as appropriate familial genetic counselling. Following this line of research, our study, IGCMU (*Identification de nouveaux Gènes Candidats à la predisposition héréditaire au Mélanome de l’Uvée*) aims to identify new candidate genes for hereditary predisposition to UM, particularly in cases not linked to BAP1 or MBD4. We will also explore the role of genes associated with other hereditary cancer syndromes and examine their potential involvement in UM, contributing to a broader understanding of the genetic landscape of this rare cancer.

## 2 Methods and analysis

### 2.1 Study design

This is a single-center interventional study on the genetic constitutional analysis of UM patients. The intervention involves blood and mouth swabs collection for genetic analysis, coupled with consultation with oncologists-geneticists.

### 2.2 Coordination

Centre Jean PERRIN (Clermont-Ferrand, France) is the sponsor of the study. The clinical research department is responsible for trial management (regulatory and financial aspects) and data management. All the data analyses will be conducted in the oncology-genetics department.

### 2.3 Objectives and outcomes

#### 2.3.1 Primary objective and outcome measure

The main objective is to identify new candidate genes for hereditary cancer predisposition among patients with uveal melanoma. The variants of interest will be selected from the data using the following filters: variants with a frequency <1% (GnomAD), if at least 2 affect the same gene; truncating or missense from a list of cancer genes; and a combined annotation-dependent depletion (CADD) score > 20 (COSMIC Tier1 and Tier2). The variants will then be interpreted using various databases and prediction tools.

#### 2.3.2 Secondary objective and outcome measure

The secondary objective is to explore genes known to be involved in predisposition to other cancers already described in relation to the occurrence of uveal melanoma, but for which the association has not yet been established with certainty.

The outcome of this objective will be measured from the number of patients with a mutation on the BRCA1, BRCA2, CHEK2, PALB2, POT1, MSH6, MLH1, SMARCE1, TP53AIP1, MLH2 and PMS2 genes.

### 2.4 Setting and eligibility criteria

Patients will be eligible to participate in the study if they meet the following selection criteria:

The inclusion criteria will be:

- Age ≥ 18 years
- Patients with a personal history of uveal melanoma (newly diagnosed, undergoing treatment, or in follow-up)
- Affiliation to the social security system or qualifying for cover by the system

The exclusion criteria will be:

- Pathogenic causal variant identified in BAP1 or MBD4
- Patients not consenting to constitutional genetic analysis for diagnostic purposes
- Patients not consenting to constitutional genetic analysis for research purposes
- Pregnant or breastfeeding women
- Patients under guardianship or curatorship

To participate in the IGCMU study, patients meeting the selection criteria will be invited in two distinct ways: either by a participating clinician who is managing their uveal melanoma, or by the French national association of patients with eye cancers (A.N.P.A.C.O.), who will present the study to its members via a presentation letter.

Individuals interested can directly contact the principal study investigator by email to apply for inclusion, or by calling the Centre Jean PERRIN oncology-genetics department. For each patient interested, a consultation with an oncologist-geneticist from the Centre Jean PERRIN oncology-genetics department will take place.

### 2.5 Intervention and participant timeline

The study will follow a structured approach divided into two main phases: a recruitment phase and a genetic-analysis phase.

During the recruitment phase, patients diagnosed with UM and their family members (if applicable) will be approached for inclusion. Recruitment will take place over a period of 12 months. The geneticists and oncologists involved in the study will manage patient inclusions and organize sample collection with the help of the clinical research department.

Once consent is obtained, two types of biological samples will be collected from each participant: a blood sample (EDTA tube) and a mouth swab (FTA card). These samples will be taken either at Centre Jean PERRIN by clinical nurses or, if necessary, at the patient’s home by a private nurse. In the latter case, pre-paid envelopes will be provided to facilitate the shipment of the samples to the Centre’s molecular oncology-genetics laboratory.

During the genetic-analysis phase, the samples collected will undergo DNA extraction, library preparation, capture, and sequencing using the NextSeq Illumina platform.

Additionally, an enrichment analysis will be performed to determine whether any variants identified are associated with specific molecular pathways. Bioinformatics analyses will be performed by a dedicated bioinformatics technician attached to the study.

The expected total duration of the study is 18 months. After the first consultation, a second consultation can be arranged if necessary for further discussions of results.

### 2.6 Whole exome sequencing

Germline DNA will be extracted from peripheral blood. Kapa library preparation kit and HyperExome probes (Roche) will be used for library preparation and capture. Sequencing will be performed on a NextSeq 2000 instrument (Illumina). All steps will be performed following the manufacturer’s guidelines.

### 2.7 Bio-informatic analysis

De-multiplexing will be performed using BCLConvert Software (Illumina). Alignment will be performed on GRCh38 human genome reference using Burrows-Wheeler Aligner. Genome Analysis Toolkit (GATK), PICARD and Samtools tools will be used for base quality score recalibration and duplicate marking. Variant calling will be performed using two different tools GATK HaplotypeCaller and FreeBayes. Annotations will be performed using Ensembl VariantEffectPredictor and Annovar. Variants will be filtered for quality score ≥30, depth ≥ 30x, and present in ≥ 20% of reads.

### 2.8 Biological filters

For BAP1, MBD4 and all candidate gene of uveal melanoma, all variants whose frequency in the general population is < 1% will be analyzed. Variants will be classified according to the American College of Medical Genetics (ACMG) recommendations. In addition, if a BAP1 or MBD4 variant is identified in patients who have never had a genetic analysis, the result will be confirmed on the saliva sample and a consultation will be organized to report the results.

For all the others genes, the variants of interest will be selected from the data using the following filters: variants with a frequency <1% (GnomAD), if at least 2 affect the same gene; truncating; splice variant with a SPIP and SpliceAI predicted effect or missense with a combined annotation-dependent depletion (CADD) score > 20 from a list of cancer genes (COSMIC Tier1 and Tier2).

### 2.9 In silico analysis

Each variant of interest will be annotated and interpreted using Alamut Visual (Interactive BioSoftware), which includes splice site analysis tools (MaxEntScan) and protein function prediction tools (SIFT, Polyphen 2.0, REVEL). The variants presenting gene and protein expression in the uveal tissue will be selected, using the protein atlas database (proteinatlas.org). The function of each gene of interest will be interpreted using Genecards (genecards.org), Uniprot (uniprot.org) and Pubmed (https://pubmed.ncbi.nlm.nih.gov/). Proposed splice site variants will also be analyzed using SPIP and spliceAI software. Differential gene expression between tumor and healthy uveal samples will be explored using GEPIA and Expression Atlas databases.

In addition, an enrichment study will be carried out to determine whether the variants identified by the previously applied filters can be linked to the same molecular pathway. Molecular pathways are already defined by online databases (PANTHER, KEGG, *etc.*). The enrichment and associated statistical calculations are carried out on the basis of a list of genes entered into an on-line computer tool (for example https://www.webgestalt.org/, https://biit.cs.ut.ee/gprofiler/gost).

### 2.10 Sample size

Since uveal melanoma is a very rare condition and given the study design, no minimum number of patients to include has been defined. Recruitment of approximately 50 patients is a reasonable estimate of the recruitment capacity.

### 2.11 Data-management

All clinical and genetic data will be collected from the patients’ medical records or obtained during consultations with the oncology and genetics teams. The data collected will be limited to what is necessary to meet the specific objectives of the study, including:

- Personal history of cancer
- Family history of cancers, including a genealogical tree
- Anatomical pathology reports related to the patients’ tumoral pathology
- Reports of constitutional and tumor genetic analyses performed on the patients, particularly those related to the occurrence of uveal melanoma
- Reports on genetic analyses related to hereditary cancer risks, if applicable
- Whole exome sequencing data in VCF format

The data capture for the study will be performed by oncologists-geneticists from the Centre Jean PERRIN Molecular Oncology and Genetics Laboratory. The data will be recorded in an Excel file specific to the study. Individuals who will have access to the data include investigators, project managers, and bioinformatics technicians. These are authorized professionals bound by professional confidentiality, and all data will be made anonymous to ensure confidentiality.

### 2.12 Trial status

Patient recruitment for the IGCMU trial is currently on-going and has been so since October 2024.

## 3 Discussion

This study aims to explore the hereditary aspects of UM by identifying new candidate genes involved in cancer predisposition. While the roles of BAP1 and MBD4 are already known in family clusters, around 78% of UMs are not linked to them (10). This highlights the need to know what other genes could have an impact on the occurrence of this disease. In order to better understand the mechanisms behind UM and improve patient outcomes, it is essential to continue research in this area. Many research teams are or have been working on this topic. In France, we can cite the Curie Institute and the Melanoma Patient Network Europe, which focus on understanding why some European individuals are at a significantly higher risk and why some patients develop aggressive forms of the disease with a high likelihood of metastasis. This project has obtained American funding under the “Rare Melanomas Focused Program Award” call for proposals with the aim of developing three themes: 1- identifying UM predisposition factors, 2- establishing and identifying isogenic cell lines from the original uveal melanocyte, and 3- identifying recurrent, immunogenic neoantigens specific to UM (15).

One study (NCT05179174) is currently recruiting UM patients in order to identify genetic and epigenetic biomarkers in UM using miRNA analysis, while also focusing on the GNA11 and GNAQ genes. These genes are located on chromosomes 9q21.2 and 19p13.3 and encode for proteins vital for signaling pathways in uveal melanoma (UM). Mutations in these genes have already been studied, and have been identified in approximately 80-90% of UM cases (3, 16). However, they have not been associated with patient outcomes or disease-free survival (17, 18).

One of the limits of our study is the small sample size. We have defined our recruitment capacity according to the number of cases identified in the French national association of patients with eye cancers (A.N.P.A.C.O.), which supports this research. In order to expand the cohort and thank to the *Plan France Médecine Génomique 2025*, we plan to request access to the national sequencing platform data, so that we can add data from other UM patients. Indeed, on a national scale in France, all cases of uveal melanoma occurring before the age of 40 are addressed to, sequenced and stored on this platform.

The possible involvement of genes like BRCA1, BRCA2, CHEK2, and POT1, which are known to play a role in other hereditary cancers, could lead to new knowledge of UM, especially in family cases (12). By conducting exome sequencing and focusing on genes associated with various hereditary cancer syndromes, this research could expand the current understanding of the genetics of UM because of similar biological mechanisms and signaling pathways that could affect its progression. For example, a recent study found that certain mutations in genes involved in the PI3K/AKT/mTOR pathway could have a prognostic value in UM. CHEK2 and PDK1, for instance, have been shown to be associated with the development of metastases, as well as DHX9. This retrospective analysis involved 69 UM cases with a median age of 59 years, and 39% presented metastatic disease at inclusion (19). Given the small size of this cohort, a larger, prospective study could be valuable to validate these findings.

Previous studies have also explored the involvement of BRCA1 (12, 20), BRCA2 (21, 22) and CHEK2 (12). The study examining the relationship between BRCA1 and UM was retrospective. Researchers also analyzed a substantial cohort of BRCA mutation carriers and explored whether any of the 469 cases developed UM. They identified two cases of UM, a frequency higher than the typical incidence in the general population, which is about five to six cases per million inhabitants (20). These findings suggest that pursuing research in this direction could be worthwhile.

One of the studies investigating the involvement of BRCA2 was prospective, conducted in Israel, and included 30% of UM cases in the country in the 1990s. In brief, 149 patients were included, but only 4 out of the 143 assessable patients were found to carry BRCA2 mutations. This limited number of mutations reduced the study’s ability to establish a strong association between this gene and UM (21). The other study exploring the involvement of BRCA2 in UM was also prospective and conducted in the 1990s. It reached a similar conclusion; however, the screening of patients could have been biased, as genomic analyses were preferentially performed on UM cases with a family history of breast or ovarian cancer — cancers that are known to be associated with BRCA mutations (22).

Other genomic analyses found only sporadic associations with UM (2, 21, 22). Some of these studies are 20 years old and current analysis techniques could provide new insights. This is why we have initiated the IGCMU study, in an attempt to find answers to these questions and expand research by including other targets such as PALB2, POT1, MSH6, MLH1, SMARCE1, TP53AIP1, MLH2 and PMS2.

The single-center design of our study enables a close coordination between oncologists, geneticists, bioinformaticians, and clinical staff, ensuring the quality in the process of genetic analysis. This approach is important given the complexity of analyzing genetic data for such a rare condition.

Finally, the identification of new candidate genes could not only improve our understanding of the genetic origins of UM, but also lead to better clinical management for patients and their families.

In conclusion, this study aims to fill a gap in the current knowledge on hereditary predisposition to uveal melanoma. By identifying new candidate genes associated with the disease, this research has the potential to enhance patient care and ultimately improve outcomes for patients with uveal melanoma.
